# Preventing type 2 diabetes: A research agenda for behavioural science

**DOI:** 10.1111/dme.15147

**Published:** 2023-05-20

**Authors:** David P. French, Amy L. Ahern, Colin J. Greaves, Rhiannon E. Hawkes, Suzanne Higgs, Rachel Pechey, Falko F. Sniehotta

**Affiliations:** ^1^ Manchester Centre of Health Psychology, Division of Psychology and Mental Health University of Manchester Manchester UK; ^2^ MRC Epidemiology Unit, School of Clinical Medicine University of Cambridge Cambridge UK; ^3^ School of Sport, Exercise and Rehabilitation Sciences University of Birmingham Birmingham UK; ^4^ School of Psychology University of Birmingham Birmingham UK; ^5^ Nuffield Department of Primary Care Health Sciences University of Oxford Oxford UK; ^6^ Division of Public Health, Social and Preventive Medicine, Center for Preventive Medicine and Digital Health (CPD) Universitätsmedizin Mannheim, Heidelberg University Heidelberg Germany; ^7^ NIHR Policy Research Unit Behavioural Science Newcastle University UK

**Keywords:** complex intervention, diet, exercise/physical activity, obesity, prevention of diabetes

## Abstract

**Aims:**

The aim of this narrative review was to identify important knowledge gaps in behavioural science relating to type 2 diabetes prevention, to inform future research in the field.

**Methods:**

Seven researchers who have published behaviour science research applied to type 2 diabetes prevention independently identified several important gaps in knowledge. They met to discuss these and to generate recommendations to advance research in behavioural science of type 2 diabetes prevention.

**Results:**

A total of 21 overlapping recommendations for a research agenda were identified. These covered issues within the following broad categories: (a) evidencing the impact of whole population approaches to type 2 diabetes prevention, (b) understanding the utility of disease‐specific approaches to type 2 diabetes prevention such as Diabetes Prevention Programmes (DPPs) compared to generic weight loss programmes, (c) identifying how best to increase reach and engagement of DPPs, whilst avoiding exacerbating inequalities, (d) the need to understand mechanism of DPPs, (e) the need to understand how to increase maintenance of changes as part of or following DPPs, (f) the need to assess the feasibility and effectiveness of alternative approaches to the typical self‐regulation approaches that are most commonly used, and (g) the need to address emotional aspects of DPPs, to promote effectiveness and avoid harms.

**Conclusions:**

There is a clear role for behavioural science in informing interventions to prevent people from developing type 2 diabetes, based on strong evidence of reach, effectiveness and cost‐effectiveness. This review identifies key priorities for research needed to improve existing interventions.

The prevalence of type 2 diabetes continues to increase in the UK and globally, with a substantial burden for those affected, and requiring an increasingly large share of the UK's healthcare budget.^1^ Important risk factors for type 2 diabetes are behavioural, notably poor diet and lack of physical activity, and there is compelling evidence that changing these behaviours can reduce incidence in people at increased risk for developing the condition.^2^, ^3^ Furthermore, a wide range of interventions for type 2 diabetes prevention are likely to be cost‐effective, including both population level and higher intensity individual approaches targeted at high‐risk populations.^4^ Whether or not people engage with these programmes or maintain engagement is also clearly within the remit of behavioural science. Thus, there is a clear role for behavioural science in informing efforts to prevent people from developing type 2 diabetes, as supported by the detailed consideration of evidence‐based behaviour change guidance on preventing diabetes in high‐risk populations.^5^ The aim of the present narrative review was to identify important knowledge gaps in behavioural science relating to type 2 diabetes prevention, to inform future research in the field. This is one of three reviews commissioned to identify gaps in research for type 2 diabetes prevention, with the other reviews considering physiological and sociodemographic issues.

The present review considers several behavioural science issues, specifically: (a) how can approaches for the whole population be more effective, particularly across subgroups and in the longer term, (b) how can diabetes prevention programmes for people at high risk of type 2 diabetes be made more effective, and (c) should greater consideration be given to stress and other emotional determinants of type 2 diabetes than is currently the case? Across all these issues, an attempt has been made to clearly indicate what is currently known and what are the important gaps in behavioural science knowledge for future research to address.

As this is a narrative review, there were no attempts to systematically review the relevant literatures, nor to formally achieve consensus. However, the authors were invited to contribute this review by the Diabetes Research Steering Group on Prevention and Management of Diabetes UK, based on their publication track records demonstrating expertise in this area. The text and recommendations were reached through an ongoing process of discussion, email correspondence and iteration on drafts of the present review, with disagreements being over matters of detail that were fairly easy to resolve. The final set of recommendations are included in Box 1.

Box 1 **Key research recommendations to advance the behavioural science of type 2 diabetes prevention**

**Population level interventions**

- Prioritise interventions enacted at scale and over longer periods of time to enable evaluations of longer‐term effectiveness
- Build a robust evidence base as to the relative impact of population‐level (in particular, choice architecture‐style interventions) across different population groups

**Disease‐specific approach**

- Understand the relative impact on engagement and cost‐effectiveness of disease agnostic, disease‐specific and tailored/targeted prevention approaches

**Diabetes Prevention Programmes: Engagement**

- Understand what additional support optimises engagement, use and understanding of BCTs in diabetes prevention interventions
- Assess the extent to which BCT engagement and effectiveness is related to demographic characteristics using routinely collected usage data in digital diabetes prevention interventions
- Explore how a human element adds value to digital diabetes prevention interventions, the training required and the extent to which support could be digitised

**Diabetes Prevention Programmes: Maintenance**

- Improve the long‐term impact (beyond 24 months) of real‐world/pragmatic diabetes prevention interventions on weight loss, physical activity (and thereby on type 2 diabetes incidence)
- Minimise impacts of diabetes prevention interventions on health inequalities, by extending the range of people for whom long‐term weight loss can be achieved
- Establish the long‐term impact (beyond 12 months) of digital diabetes prevention and/or weight loss interventions
- Identify the most cost‐effective strategies for maintaining behaviour change, including both innovations in maintenance theory (addressing the psychosocial and physiological drivers of obesogenic behaviour) and structural maintenance approaches (such as stepped care or continuous intervention)

**Diabetes Prevention Programmes: Mechanism**

- Assess intervention fidelity of diabetes prevention interventions to establish the key active ingredients for achieving behaviour change
- Update systematic review evidence on which BCTs work in changing health behaviours (e.g. diet and physical activity) and outcomes (e.g. bodyweight and blood glucose levels), including analysis by different demographic groups
- Update systematic review evidence on what timing and dose of BCT delivery is required in diabetes prevention interventions to understand how using more or fewer techniques can achieve weight loss and reduced blood glucose levels
- Compare the variation in modes of BCT delivery in different diabetes prevention interventions to examine which modes of BCT delivery are associated with better outcomes
- Assess service user receipt of intervention content delivered via different modalities in diabetes prevention interventions to determine whether the intervention content is changing the behaviours it intended to change

**Diabetes Prevention Programmes: New approaches**

- Explore the incorporation of new strategies that enhance psychological flexibility, such as third wave cognitive behavioural therapies or cognitive training
- Understand when and how social context influences behaviour change and could be leveraged in novel ways
- Investigate the potential for just in time adaptive interventions to augment effectiveness and personalisation

**Diabetes prevention: The role of mental health**

- Understand the optimal balance between the message that type 2 diabetes is preventable to motivate people engaging in diabetes prevention, with the message that it is not a matter of personal responsibility to develop type 2 diabetes in order to reduce (self‐)stigma and associated distress
- Explore the effects of additional psychological and social mental health support in diabetes prevention
- Understand the relationships between mental well‐being and type 2 diabetes risk

## 1 UNDERSTANDING THE VALUE OF A DISEASE‐SPECIFIC APPROACH

An evidence‐based approach to considering behaviour science issues in preventing type 2 diabetes must consider which behaviours are important in increasing or reducing risk. To date, behavioural approaches to type 2 diabetes prevention have focussed on comprehensive health behaviour change interventions with the predominant focus on achieving weight loss through changes in diet and physical activity.^2^ Although there is some evidence that some behavioural changes can influence glycaemia in the absence of weight loss, weight loss appears to be the primary mechanism of effect. The content of diabetes prevention programmes (DPPs) is often indistinguishable from behavioural weight management interventions^6^ and prevention programmes for other non‐communicable diseases such as cardiovascular disease and cancer.^7^ This raises the question of to what extent disease‐specific‐advice about behaviour change is critical to the effectiveness of and engagement in DPPs.

Evidence to date does not support disease‐specific diet or physical activity recommendations for type 2 diabetes prevention.^8^ We do not rule out the potential for future studies to generate new insights, but current evidence supports similar recommendations to the general population. The salience of being identified as at risk of type 2 diabetes could motivate engagement, but this risk is not always well understood.^9^ Combining prevention programmes and highlighting potential benefits across multiple diseases might be more compelling for all patient groups and encourage greater engagement.^10^ There is some evidence that intervening earlier in the disease trajectory and offering interventions to all those with elevated body mass index might have greater population health benefits than focussing only on those with non‐diabetic hyperglycaemia.^11^ Future research should evaluate the impacts of such an approach on engagement, effectiveness and cost‐effectiveness compared with current disease‐specific prevention approaches and more targeted/tailored approaches.

## 2 HIGH RISK VS POPULATION APPROACHES

Upstream population‐level approaches aim to improve health across the whole population (regardless of individuals' level of risk), acting as a preventative measure by targeting underlying causes of disease.^12^ High‐risk strategies, by contrast, focus efforts on identifying and intervening to improve the health of those most likely to experience disease burden. Given the high‐risk approach does not attempt to change the incidence of individuals at high risk of type 2 diabetes, changing upstream causal factors is a necessary complementary strategy.^13^, ^14^ This is particularly critical against the current backdrop of increasing prevalence of diabetes in the UK,^1^ placing an increasing burden on prevention programmes targeting this high‐risk group of individuals.

Measures tackling upstream population‐level determinants of behaviour can take many forms. These include fiscal regulations (such as the UK Soft Drinks Industry Levy), media campaigns (e.g. Change4Life) or micro‐environmental (or choice architecture/‘nudging’) interventions (changing physical elements of settings, for example, changing the layout of a supermarket).

### 2.1 Equity of impact from environmental interventions across population groups

Population approaches that require little agency (need for personal engagement) have been hypothesised to be less likely to increase health inequalities.^15^, ^16^ In particular, micro‐environmental interventions – often characterised as relying largely on non‐conscious processes^15^, ^17^ – have been highlighted as potentially being more likely to work equitably across the population. This hypothesis remains largely untested.

To date limited conclusions can be drawn from systematic reviews on micro‐environmental interventions as to whether demographic characteristics might moderate intervention effectiveness, largely due to a lack of studies reporting such information.^18^ One hypothesis is that moderation depends on the degree to which an intervention is information based (or cognitively oriented), with a review of socioeconomic inequalities in the impact of healthy eating interventions concluding information‐based interventions tended to be more effective for individuals with higher socioeconomic position (SEP).^19^ In contrast, no identified environmental change interventions were likely to lead to differential impact by SEP.^19^ However, this review only included a small number of studies, and the literature in this area has expanded widely in the past decade. More recent reviews looking at impact by SEP have provided somewhat contradictory evidence.^20^, ^21^

As such, there is a need for large studies and/or meta‐analyses of existing data^22^ that explore effectiveness of micro‐environmental interventions by a wider range of demographic characteristics, to provide a robust evidence base as to whether these interventions are (as) impactful across different population groups. If interventions are more effective for those groups with, for example, higher education, then health inequalities may be exacerbated as a result.

### 2.2 Long‐term impacts of population‐level approaches

Type 2 diabetes, like other chronic diseases, develops over several years. In the same period, an individual will, for example, make countless dietary and physical activity selections, effects of which may aggregate and interact with other risk behaviours.^23^ If an intervention is to make a longer‐term impact, at minimum its implementation needs to be sustained over time. Yet, many micro‐environmental interventions have been implemented over a relatively short timescale. As a first step towards providing evidence for a pathway to longer‐term impact, such interventions need to be designed, implemented and evaluated for an extended duration, and the relative effectiveness of these interventions over time assessed. Where feasible, accurately identifying longer‐term economic impacts of environmental or population level interventions could feed into improved modelling of outcomes.^14^ This would help to compare such interventions with the benefits of more targeted high‐risk individual‐level approaches.

One key context for such interventions is supermarkets, where a substantial proportion of dietary decisions are made and where a range of interventions (targeting economic, education and store environmental features) has been tested to identify strategies, which may support dietary change.^24^ However, in the small proportion of studies conducted in supermarkets (rather than hypothetical or virtual settings), these interventions have been tested in a small number of stores, on a small number of products, and for relatively short durations. While this may in part reflect the competitive economic environment in which supermarkets operate, which may discourage retailers from moving alone, there are opportunities for effective collaboration with industry. Identifying interventions that can be enacted at scale and over longer periods of time would facilitate evaluations of both potential moderators of intervention impact and intervention effectiveness over time.

## 3 DIABETES PREVENTION PROGRAMMES

Although population approaches have many appealing features, to date there has been much more focus on high‐risk approaches, which involve identifying those individuals who are at increased risk of developing type 2 diabetes. Those individuals can then be offered services to prevent disease progression, which are generically known as Diabetes Prevention Programmes (DPPs). There is strong evidence from multiple international trials that DPPs are effective at preventing progression to type 2 diabetes,^3^ and some evidence from routinely collected data in England that DPPs can prevent progression outside of a trial context.^25^ Despite this, there are several uncertainties about how to increase the effectiveness of these DPPs.

### 3.1 Engagement in Diabetes Prevention Programmes

Although the effectiveness of DPPs has been established, the population impact on type 2 diabetes prevention crucially depends also on reach into the population at high risk. There has been considerable variation internationally,^26^ with the National Health Service Diabetes Prevention Programme (NHS‐DPP) in England one of those that achieved better reach, with over 500,000 people being referred within four years of the programme being implemented nationally.^25^ Relatedly, to avoid exacerbating inequalities, it is essential that DPPs are taken up by minority ethnic groups and people of lower SEPs. There is clear evidence that development of type 2 diabetes is socially patterned, with risk of type 2 diabetes being higher in many ethnic minority groups, especially people from South Asian and black Caribbean background.^27^ Further, management of the condition in those who develop the condition is worse in those of lower SEP, for example, those with lower incomes.^27^ The NHS‐DPP has tended to disproportionately enrol older people, but fewer of those with lower SEP.^28^

The introduction of digital versions of the NHS‐DPP may help with reaching some groups that a face‐to‐face version does not reach, for example, a younger population, or those whose work preventing them regularly attending face‐to‐face group sessions. Research on the digital NHS‐DPP has found that people on the digital programme have increased engagement with key intervention components such as setting goals and monitoring their health behaviours when they receive interaction with a health coach associated with those components.^29^ This programme of work has found that health coaches in the digital NHS‐DPP help to increase engagement, motivation and accountability for service users on the programme.^30^ More research is needed to better understand the amount of health coach support required to keep users engaged in these digital programmes and how this support is best delivered (e.g. via telephone, messaging).

At the time of writing, people are given a choice to sign up to the face‐to‐face or digital NHS‐DPP, either via general practitioner referral or self‐referral. It is likely that programmes that require people to identify need, seek out how to sign up and retain engagement are likely to exacerbate inequalities as people with low numeracy, literacy and health literacy, and fewer financial and other resources tend not to engage with programmes compared with less disadvantaged groups.^15^ Given all this, there is a need to identify how to better engage with those groups that are not engaging with programmes such as NHS‐DPP at each stage. Detailed monitoring of precisely which groups are engaged at invitation, enrolment and completion stages for both face‐to‐face and digital versions of the NHS‐DPP is needed, as well as the reasons for differential engagement.

This understanding of reasons for lack of engagement should be used to develop approaches, which may increase uptake of those ethnic minority and lower SEP groups that are not engaging.^9^ The use of qualitative methods to understand reasons for people not taking up the offer of DPPs would be useful, especially in ethnic minority groups. Further, it appears worthwhile to consider co‐producing alternative offers of DPPs to those groups who typically choose not to take up offers of DPPs and/or to consider whether recommendations by particular trusted individuals (e.g. general practitioners) produces greater uptake.

Further examination of exactly what elements of digital DPPs people choose not to engage with and what content is offered immediately before people at high risk disengage from digital DPPs should shed light on reasons for lack of engagement in the full programme. The delivery of the NHS‐DPP by multiple providers allows comparison of uptake and engagement patterns between providers,^28^ and further consideration of why there are these differences appears warranted.

Finally, given that a ‘one size fits all’ DPP is unlikely to meet all needs of people with type 2 diabetes, it may be worth considering further formats for delivery. That is, a briefer version may be more acceptable to people who would not be willing to engage in a 9‐month programme. Alternatively, for those whom the current DPP does not produce change, a more in‐depth version might be useful. Clearly, all options have resource implications, rendering alternative programmes feasible only when qualitative and quantitative data provide a good a priori case. Evaluations of which alternatives are most cost‐effective and for whom are therefore needed.

### 3.2 Maintenance of behaviour change

Long‐term follow‐up of clinical diabetes prevention trials suggests that relatively small amounts of weight loss (as little as 1.5–2 kg) seem to be sufficient to substantially reduce diabetes incidence in people at risk of type 2 diabetes,^3^ and these levels of weight loss are achievable by real‐world DPPs.^31^ However, weight loss is typically followed by a slow regain in weight, and as weight is regained so is glycaemia.^32^ While cumulative incidence of diabetes is reduced despite this regain, it is likely that the long‐term benefits to individual health and health economies from DPPs would be much greater if longer‐term changes in behaviour (e.g. diet and physical activity) and in weight could be achieved.^33^ Notably, despite promising initial evidence on effectiveness of digital programmes, there is a need for high quality trials to establish the long‐term impact (beyond 12 months).^34^

With regards to type 2 diabetes, there is only limited evidence on the long‐term effects (beyond 12 months) of interventions on physical activity type 2 diabetes.^35^ The most recent example (the PROPELS trial) showed that changes in accelerometer‐assessed walking activity (532 steps per day) at 12 months were not sustained at 48 months.^36^ In the wider adult population, a recent systematic review of long‐term changes to physical activity following interventions indicates that effects are sometimes sustained, although there are again trials reporting effects beyond 12 months.^35^ More research is needed to understand what types of interventions support sustained physical activity, for whom, and under what circumstances. Different interventions may also be needed depending on the type of activity targeted.

Current approaches to address long‐term maintenance of weight loss or physical activity have been limited in scope/theoretical diversity, focusing mainly on behavioural self‐regulation (action‐planning, self‐monitoring and reviewing of progress) and more generic ‘problem‐solving’ techniques.^37^ It is not clear how well such interventions (as well as the preceding behaviour change programmes) prepare people sufficiently for transitioning to self‐maintenance and develop healthy habits. Some have suggested that approaches that focus more on needs‐satisfaction and management of the psychological, social and physiological drivers of obesogenic behaviours might be worth including in behaviour maintenance interventions.^38^, ^39^ However, to date, few such approaches have been evaluated for long‐term effectiveness.

Alternative structural/enhanced delivery approaches to maintenance, such as continuous (ongoing) intervention, regular follow‐up support, stepped care approaches, or ‘rescue‐plan’ interventions have also not been widely tested. Specifically, although the DPP that was evaluated in a trial in the United States used ongoing treatment and rescue planning,^2^ it was not translated into the ‘real world programmes’. Such trade‐offs between what increases effectiveness in trials and what is considered implementable in routine practice is an ongoing debate.^40^

### 3.3 Understanding mechanism (and context)

#### 3.3.1 Clear reporting of intervention content to optimise intervention designs

To shed light on mechanisms by which DPPs might work, a systematic review analysed the evidence on effective intervention components of behaviour change interventions for type 2 diabetes prevention.^31^ This analysis was limited by under‐reporting of intervention content, which has been a problem with interventions designed to change health behaviours for some time.^41^ Greater use of logic models or explicit description of how interventions are expected to work^42^ should be included in intervention designs to establish the key intervention techniques and mechanisms of action in DPPs.^43^

Standardised frameworks to describe intervention content should be encouraged, such as the Template for Intervention Description and Replication (TIDieR) checklist.^41^ At least one or more detailed method of reporting intervention content such as the Behaviour Change Technique Taxonomy^44^ could be used to describe the active behaviour change components so that interventions can be understood and replicated by others. Researchers in health can often be faced with rigid article structure and strict word limits imposed by journals, which may produce barriers to reporting complex interventions in sufficient detail. However, without this clear description of intervention rationale and behaviour change content, systematic reviews of type 2 diabetes prevention interventions are severely hampered in testing which behaviour change techniques (BCTs) or other intervention features work in changing health behaviours and why. To overcome this, health journals should encourage researchers to submit supplementary material clearly reporting intervention content if there is not the scope to include this detail in the main article.

#### 3.3.2 Which BCTs work in changing health behaviours for type 2 diabetes prevention and for which populations and contexts?

Current NICE guidance recommends self‐regulation BCTs (e.g. goal setting, self‐monitoring) to produce behaviour change in people at high risk of type 2 diabetes.^5^ Systematic reviews have also more recently suggested self‐regulatory BCTs to be effective in changing dietary and physical activity behaviours to reduce HbA1c and weight^45^ and in technology‐driven type 2 diabetes prevention interventions.^34^ However, there is a lack of evidence on how two or more BCTs may interact to improve health behaviours, in addition to the timing and ‘dose’ of BCT delivery required to produce changes in outcomes. It is possible that interventions containing more strategies to help people change their diet and physical activity behaviours may be more effective,^34^, ^46^ but more evidence is required to understand how using more or fewer BCTs in type 2 diabetes prevention interventions can impact outcomes.

An update to the existing review of moderators of DPP effectiveness may be able to address this, though such reviews are dependent on the clear reporting of intervention content and outcomes in studies.^31^ It is also important for studies to measure intervention fidelity going forward (i.e. the extent to which the intervention components are delivered as intended). Without a fidelity assessment, it cannot be ascertained whether intervention (in)effectiveness is due to inherent intervention features or factors added or omitted during implementation.

It is also important to consider that much research regarding what BCTs are most effective at producing behaviour change included in guidance such as the NICE guidance^5^ is produced in different populations than those that are typically enrolled in DPPs. Notably, the research is typically performed with younger, more educated and ethnically homogeneous samples. Such samples may struggle with the self‐regulation content that DPPs typically use, such as goal setting, self‐monitoring and planning.^47^ By contrast, qualitative research with service users on the NHS‐DPP revealed that understanding of some key BCT content (e.g. action planning, problem solving) was frequently poor, with many service users having little recollection of these BCTs.^30^, ^48^ This is in line with other research which typically finds that those self‐regulation techniques that are effective with younger people have much less impact with older people.^49^ There is direct evidence from DPPs that the engagement with these BCTs is enhanced when support was provided.^48^ Further evidence is needed, both on the relationship between usage of BCTs and changes in behaviour and outcomes, and also what additional support optimises engagement and effective usage. The use of routinely collected data from digital DPPs should help with assessing the extent to which BCT engagement and effectiveness is related to demographic characteristics and also comparing usage patterns between different providers to examine how naturally occurring variations in support relate to usage.

#### 3.3.3 What are the most effective modes of BCT delivery in interventions targeting type 2 diabetes prevention?

There is limited knowledge about which modes of BCT delivery are associated with better outcomes in type 2 diabetes prevention. It is also possible that some BCTs are more or less conducive to being well understood depending on whether they are delivered in a group setting or an individualised one‐to‐one setting; for example, some BCTs may require substantial one‐to‐one support that is difficult to deliver effectively to a large group with diverse needs. One possibility is to use the variation in modes of BCT delivery in DPPs run by different providers as a natural experiment to directly examine this. Assessing service users' receipt of intervention content delivered via different modalities in DPPs would also determine whether the intervention is changing the behaviours it intended to change.

The more recent implementation of digital behavioural interventions provides potential to target type 2 diabetes prevention at scale. Research on the digital NHS‐DPP suggests a human coaching element still appears critical for service user understanding of BCT content and to increase accountability and provide person‐centred support,^30^ which has also been evidenced in digital weight loss programmes.^50^ Some BCTs appear to be well understood and suited for digital delivery without further support (e.g. self‐monitoring), but other BCTs such as goal setting, feedback and problem solving may be better understood when there is a coaching element.^30^ Future research should investigate how this human element adds value, the most effective way to deliver this behaviour change support (e.g. via telephone, messaging), what level of training is required (e.g. the ‘depth’ of behaviour change technique training^51^) and whether this support could be digitised to some extent.

#### 3.3.4 Beyond standard behaviour change techniques?

DPPs often incorporate techniques adapted from second wave cognitive behavioural therapies, such as learning to identify and change thoughts that might undermine behaviour change. Weight management interventions have begun to incorporate elements from third wave cognitive behavioural therapies (3wCBT), which in contrast to earlier forms of cognitive behaviour therapy focus on the process of thoughts, rather than their content to help people achieve behaviour change. The most promising of these are interventions that incorporate acceptance and commitment therapy (ACT).^52^ A premise of ACT is that a person's ability to enact a valued activity (e.g. eating a healthier diet) will be improved if there is non‐judgemental acceptance of uncomfortable feelings that may accompany engagement with that activity (e.g. perceived loss of enjoyment from eating a healthier diet). ACT interventions aim to help individuals act in line with their goals even in the face of such challenging emotions or feelings (often referred to as psychological flexibility). ACT interventions improve long‐term weight outcomes for people with obesity^38^ and improves glycaemia and self‐care for people with type 2 diabetes.^53^ Future studies should evaluate the effectiveness of incorporating such 3wCBT into type 2 diabetes prevention interventions and consider how this may be done in a scalable and cost‐effective manner to maximise reach and maintain efficacy.

Psychological flexibility can also be enhanced via cognitive training techniques aimed at improving executive functions such as working memory and cognitive control that underpin reflective food choices.^54^ There is limited evidence, mainly from healthy weight samples that training of cognitive control improves eating behaviours^55^ and some evidence that cognitive training in the context weight management is associated with weight loss.^56^ Computerised training has been reported to improve memory function in people with type 2 diabetes, but this improvement did not translate into improvements in measures‐related self‐care.^57^ Training was associated with reduced fat intake but only for participants who were already motivated to alter their diet. Moreover, as with other cognitive training trials, the training was perceived as difficult.^57^ Cognitive training could be a cost‐effective adjunct to behavioural treatment for type 2 diabetes prevention, but further research is required to refine delivery and engagement and identify individuals, who may benefit most from training.

Individual behaviour change tends to be at the forefront of DPPs, but it is also recognised that social contexts and personal relationships have an influence on an individual's behaviour^58^ and that interventions that include members of a social network can improve outcome in patients with type 2 diabetes.^59^ The fact that family members, friends and peers can provide emotional and practical support for loved ones who are trying to make behavioural changes is often highlighted as key underlying mechanism. However, there are various other ways in which members of a social network can affect the outcome of an intervention both positively and negatively. For example, a family member or partner can influence dietary choices of someone at risk of type 2 diabetes via modelling of healthy behaviours, which encourages uptake of the modelled behaviour both through a desire to please as well as unconscious mimicry of the modelled behaviour.^60^ However, in some relationships, a partner or spouse may be more likely to use social control to coerce behaviour, which may be counterproductive.^61^ Within a group context, people use social comparisons to evaluate their own behaviour. Whereas upward social comparison (comparing oneself with another similar person who is performing much better than oneself) can increase motivation for change, self‐efficacy and self‐esteem,^62^ downward social comparison can lead to reduced motivation to continue with behaviour change because of a feeling that one is doing well relative to others. These data suggest that consideration of the social context of a prevention intervention is important, but they also highlight the multifaceted nature of the social dynamics within close social relations and peer support groups. Future research could be directed at understanding when and how social contacts either facilitate or undermine behaviour change^63^ so that the potential of social context to assist in type 2 diabetes prevention can be leveraged and more effective socially grounded interventions can be developed.

The COVID‐19 pandemic has accelerated a move towards digital delivery of DPPs, but to date this has predominantly focussed on translating traditional interventions into digital formats. Powerful advances in smartphones and wearable technology could be harnessed to better understand the antecedents and consequences of behaviour and to implement innovative intervention approaches. Ecological momentary assessment can enable us to model how variation in momentary cognitive and emotional states might relate to behaviours and interact with contextual factors.^64^ This can inform just in time adaptive interventions (JITAIs), which can deliver tailored behaviour change support to people when they most need it, such as when they are most at risk of engaging in behaviour that contrasts with their health goals.^64^ Importantly, both tailoring and delivery of support can be initiated by the application in response to data collected during the intervention, without direct action from the user. This work is in its relative infancy and to date no studies have specifically focussed on JITAIs for type 2 diabetes prevention, but early work in related fields of weight management^52^ and physical activity promotion^65^ is promising and their potential in type 2 diabetes prevention should be explored.

## 4 THE ROLE OF MENTAL HEALTH

People with type 2 diabetes are more likely to experience poor mental health than people without diabetes. The unmet need for more emotional and psychological support has been acknowledged elsewhere.^66^

A number of psychiatric disorders have been associated with increased risk of type 2 diabetes.^67^ Higher prevalence of depression and anxiety is evident in people at risk of developing type 2 diabetes,^68^ and there is increasing evidence that depression and anxiety are predictive of transition to diabetes.^69^ Eating disorders, particularly binge eating disorder, are more prevalent in people with type 2 diabetes than the general population and are associated with increased risk of developing type 2 diabetes. However, DPPs rarely measure eating disorder outcomes and do not address diagnosis and treatment of eating disorders. Recent articles have highlighted the importance and the challenges of improving assessment, monitoring and treatment of eating disorders in the context of weight management and type 2 diabetes,^70^, ^71^ and similar arguments can be extended to diabetes prevention. People with severe mental illness are at increased risk of type 2 diabetes, which contributes substantially to their reduced life expectancy. Few high‐quality studies have examined diabetes prevention in people with SMI and those that have suggest effects are more modest than in the general population and that tailoring may be need to address their specific challenges.^72^ Further research is needed on what behavioural strategies and modes of delivery are most effective in people with SMI.

Whilst mental health has become a focus in diabetes research on its own right, there are likely important relationships to behavioural factors in and around type 2 diabetes prevention which warrant further investigations. For many, the diagnosis of pre‐diabetes follows years of failed efforts to lose weight and maintain weight loss. The experience of high personal priority for weight loss and/or type 2 diabetes prevention with associated lack of success is likely to come with emotional costs.^73^ This might be further accelerated through external or internalised stigma from the perception that type 2 diabetes is preventable, a point which is further emphasised by the provision of an evidence‐based national DPP.

Poor mental health is associated with executive dysfunction and undermines the ability to change behaviour. Unmet emotional needs are a key barrier for weight loss and weight loss maintenance.^74^ For example, adherence to physician recommended lifestyle changes in people at risk of type 2 diabetes is lower when people report more depressive symptoms.^75^ Consequently, participants in DPPs with higher depressive symptom burden at baseline and change over the programme benefit less from such programmes in terms of behaviour change.^76^

Type 2 diabetes prevention offers the possibility to delay or avoid type 2 diabetes within a population with increased psychological distress. With this possibility comes risk of failure, which may in turn increase anxiety and distress. There is a pressing need to identify the optimal provision of mental health support in DPPs to address the complex need of the population of people living with pre‐diabetes. Whilst some DPPs appear to show beneficial effects on mental health,^77^ DPPs may need an explicit account on how behaviour change and mental health aspects are balanced and for whom.

## 5 CONCLUSIONS

There is a clear role for behavioural science in informing interventions to prevent people from developing type 2 diabetes. Current protocols for DPPs, based on protocols from 20+ years ago, are effective and there have been successes in scaling these. However, there is a need to further optimise these interventions based on evidence and to find ways to maximise inclusivity, especially in relation to sub‐groups defined by age and ethnicity. Moreover, there is a need to think creatively about new approaches to sustained behaviour change that meet the needs of a wide range of people. It is important that future research considers interventions that more fully address emotional, cognitive and social influences on behaviour. Finding ways to improve the effectiveness, reach and cost‐effectiveness of lifestyle interventions to prevent type 2 diabetes would also inform future lifestyle interventions for diabetes management.

## CONFLICT OF INTEREST STATEMENT

ALA is on the scientific advisory board for WW (formerly WeightWatchers). All other authors declare no conflicts.
